# Inverse relationship between microRNA-155 and -184 expression with increasing conjunctival inflammation during ocular *Chlamydia trachomatis* infection

**DOI:** 10.1186/s12879-016-1367-8

**Published:** 2016-02-03

**Authors:** Tamsyn Derrick, Anna R. Last, Sarah E. Burr, Chrissy h. Roberts, Meno Nabicassa, Eunice Cassama, Robin L. Bailey, David C. W. Mabey, Matthew J. Burton, Martin J. Holland

**Affiliations:** 1 Faculty of Infectious and Tropical Diseases, London School of Hygiene and Tropical Medicine, London, United Kingdom; 2 Disease Control and Elimination Theme, Medical Research Council Unit The Gambia, Fajara, The Gambia; 3 Programa Nacional de Saúde de Visão, Ministério de Saúde Publica, Bissau, Guinea Bissau

**Keywords:** Chlamydia trachomatis, Trachoma, MiRNA, Inflammation, RNAseq, MiR-155, MiR-184, Follicular trachoma

## Abstract

**Background:**

Trachoma, a preventable blinding eye disease, is initiated by ocular infection with *Chlamydia trachomatis* (Ct). We previously showed that microRNAs (miR) -147b and miR-1285 were up-regulated in inflammatory trachomatous scarring. During the initial stage of disease, follicular trachoma with current Ct infection, the differential expression of miR has not yet been investigated.

**Methods:**

Conjunctival samples were collected from 163 children aged 1–9 years old living in a trachoma-endemic region of Guinea Bissau, West Africa. Small RNA sequencing (RNAseq) was carried out on samples from five children with follicular trachoma and current Ct infection and five children with healthy conjunctivae and no Ct infection. Small RNAseq was also carried out on human epithelial cell lines infected with ocular Ct strains A2497 and isogenic plasmid-free A2497 in vitro. Results were validated by quantitative PCR (qPCR) in 163 clinical samples.

**Results:**

Differential expression of RNAseq data identified 12 miR with changes in relative expression during follicular trachoma, of which 9 were confirmed as differentially expressed by qPCR (miR-155, miR-150, miR-142, miR-181b, miR-181a, miR-342, miR-132, miR-4728 and miR-184). MiR-155 and miR-184 expression had a direct relationship with the degree of clinical inflammation. MiR-155 was up-regulated (OR = 2.533 ((95 % CI = 1.291–4.971); *P* = 0.0069) and miR-184 was down-regulated (OR = 0.416 ((95 % CI = 0.300–0.578); *P* = 1.61*10^−7^) as the severity of clinical inflammation increased. Differential miR expression was not detected in HEp-2 or HCjE epithelial cells 48 h post infection with Ct in vitro. HCjE cells, a conjunctival epithelial cell line, had a markedly different miR background expression compared to HEp-2 cells.

**Conclusions:**

In follicular trachoma, expression of miR-155 and miR-184 is correlated with the severity of inflammation. This likely reflects host regulation of the immune response and a prolonged period of wound healing following the clearance of Ct. Prolonged healing may be associated with subsequent development of scarring trachoma.

**Electronic supplementary material:**

The online version of this article (doi:10.1186/s12879-016-1367-8) contains supplementary material, which is available to authorized users.

## Background

Trachoma is the leading infectious cause of blindness worldwide and is initiated by infection of the conjunctival epithelium with the obligate intracellular bacterium *Chlamydia trachomatis* (Ct). In trachoma-endemic areas children suffer repeated episodes of infection and the effects of the associated immune response, which is characterized by a follicular conjunctivitis. In some individuals this triggers a chronic inflammatory response, causing scarring of the eyelid over the course of the lifetime of the individual, often in the absence of detectable Ct infection [1]. Conjunctival fibrosis causes entropion (in-turning of the lid margin) and subsequent trichiasis, the inward turning of eyelashes, which scratch the cornea causing mechanical damage leading to blinding corneal opacification in a proportion of cases. The mechanisms of fibrotic progression in the absence of Ct infection and the reason why only some individuals in trachoma-endemic communities develop scarring despite similar exposure to infection are unknown.

Trachoma is endemic in 51 countries and is associated with visual impairment in 2.2 million people worldwide (of whom 1.2 million are irreversibly blind) [2]. The World Health Organization (WHO) strategy for the elimination of trachoma (“SAFE”) consists of mass distribution of antibiotics (MDA) to communities to treat infection, surgery for trichiasis, promotion of facial hygiene and environmental and sanitation improvements such as provision of latrines to reduce transmission [3].

Whilst MDA is generally effective, there are concerns regarding its efficacy in hyper-endemic settings such as Ethiopia, where trachoma prevalence remains high despite >5 years of biannual MDA in some districts [4]. High recurrence rates of trichiasis following lid margin rotation surgery [5] and the presence of follicular trachoma and incident scarring in populations where Ct infection prevalence has been controlled [1, 6] are also of concern. In order to develop an anti-Ct vaccine that invokes a protective immune response and to design therapeutic treatments to halt progressive scarring, a better understanding of the immunopathology of chlamydial disease is required.

Experimental conjunctival Ct infection of human volunteers found the incubation period to be between 2 and 19 days post inoculation [7, 8], whilst mathematical models based on data from disease and infection studies in trachoma-endemic communities estimated this period as around 17 days [9]. Although some infected individuals remain asymptomatic, many will develop clinical signs of follicular trachoma, such as lymphoid follicles and papillary inflammation. The median duration of follicular trachoma has been observed to decrease with age; from 13.2 weeks in 0–4 year olds to 1.7 weeks in individuals of 15 years or more [10]. The cumulative incidence rate of follicular trachoma is also reduced threefold with age, implying that some degree of protective immunity is acquired [10]. There is a lag time between the clearance of infection and the disappearance of clinical signs of follicular trachoma on both an individual (median time of five weeks [9]) and population level [11, 12].

MicroRNA (miR) are small single-stranded RNA species that regulate gene expression post-transcriptionally. MiR can have profound effects on health and disease and there are many examples in which abnormal expression of a single [13] or small number [14] of miR are associated with disease. We have previously found that miR-147b and miR-1285 are up-regulated in adult cases of scarring and inflammatory trachoma [15]. Others have investigated the miR response to Ct and *C. muridarum* (Cm) in the murine genital tract [16–18], but the miR response to Ct in human disease has not yet been reported. In this study we examine the miR response in clinical cases of follicular trachoma with Ct infection and during Ct infection of cultured human epithelial cells.

## Methods

### Ethics, consent and permissions

The study was conducted in accordance with the declaration of Helsinki and was approved by the Ethics committee of the London School of Hygiene and Tropical Medicine (reference: 6433) and the Comité Nacional de Ética em Saúde of Guinea Bissau (reference: 012/CNES/INASA/2013). Written informed consent was obtained from a parent or guardian before participants were enrolled in the study.

### Clinical sample collection

Prospective samples were collected from children aged from 1 to 9 years residing on seven islands of the Bijagos Archipelago, Guinea Bissau, 6 islands of which were trachoma treatment naïve at the time of sample collection. Children were screened for clinical signs of trachoma by a trained clinician using the WHO 1981 FPC scoring system [19]. Individuals presenting with follicular trachoma scores of “F2” or “F3” were roughly equivalent to “Trachomatous Inflammation − Follicular (TF)” in the WHO simplified trachoma grading system [20] and are hereafter referred to as TF cases. Individuals with pronounced papillary hypertrophy and diffuse infiltration (score “P3“) were equivalent to “Trachomatous Inflammation − Intense (TI)” in the WHO simplified grading system. Individuals with no clinical signs of follicles (F0), papillary hypertrophy (P0) or conjunctival scarring (C0) were classed as normal healthy controls (N). A case was defined as a child with TF in both eyes irrespective of P score. A healthy control was enrolled for each TF case where a case was defined as an individual with an F-score ≥ F2. Cases and controls were matched by age, gender and village.

Two conjunctival samples were taken from the left upper tarsal conjunctiva of each participant with Dacron swabs (Puritan, Guilford, ME, USA) using standard methodology [21, 22]. Swabs were stored in RNAlater (Ambion, Life technologies, Carlsbad CA, USA) and kept in a cold chain in the field before subsequent storage at −80 °C. The two swabs from each individual were pooled for RNA and DNA extraction.

### In vitro culture and Ct infection of human epithelial cell lines

Human epithelial type 2 (HEp-2) cells were cultured in Minimum Essential Medium (MEM) (Gibco, Life Technologies) supplemented with 1 % gentamicin (Gibco), 10 % fetal bovine serum (FBS) (Gibco), and 1 % L-glutamine (Gibco). For the in vitro experiments, cells were seeded in 12 well plates at 3×10^5^ cells per well in MEM. An immortalized human conjunctival epithelial cell line (HCjE) was cultured in Keratinocyte serum free medium (K-sfm) (Gibco) supplemented with 25 μg/ml bovine pituitary extract (Gibco), 0.2 ng/ml epidermal growth factor (Gibco), 0.4 mM CaCl_2_ (Sigma, St. Louis, MO, USA) and 5 % gentamicin and incubated at 37 °C with 5 % atmospheric CO_2._ For experiments, HCjE cells were seeded in 12 well plates at 3×10^5^ cells per well in 1:1 K-sfm [with supplements]: Dulbecco’s Modified Eagle Medium: Nutrient Mixture F12 (DMEM/F12) [with 10 % FBS, 1 % gentamicin and 1 % L-glutamine] (Gibco) and were switched to DMEM/F12 [with supplements] upon infection 24 h later.

HEp-2 and HCjE cells were infected with 3 different ocular-derived serovar A Ct strains: A2497 (described in [23]), its plasmid cured counterpart (A2497P-) [24] and A/HAR-13 [23]. Cells were infected 24 h after seeding at a multiplicity of infection (MOI) of 1 and without the addition of cycloheximide. Ct cultures and mock-infected cells were centrifuged at 1800 r.p.m. for 1 hr to facilitate infection and were then incubated at 37 °C in 5 % CO_2_. Six replicates were carried out for each biological condition. At 48 h post-infection (hpi), media was removed and cells were washed in 1X phosphate-buffered saline (PBS) (Sigma). Productive infection was measured by digital PCR, as detailed below and through visualization of inclusions using confocal microscopy.

### Microscopy

Cell monolayers were seeded on glass coverslips and at 48 hpi media was removed and cells were washed in 1X PBS. 200 μl ice cold 100 % methanol was added to each well for 10 min. In order to block the cells, they were washed in 1X PBS and incubated in 200 μl 1 % w/v bovine serum albumin (BSA)/PBS for 30 min at room temperature. Cells were then incubated for 30 min in the dark with 100 μl of a 1 % v/v solution of anti-Chlamydia trachomatis MOMP antibody (FITC) (ab30951, Abcam, Cambridge, UK) in 1 % w/v BSA/PBS. Cells were washed 3x in 1X PBS for 5 min each wash. DAPI dilactate (1 μg/ml (Sigma)) was added for 5 min. Cells were washed 3x in 1X PBS. Slides were mounted with coverslips and viewed on a Zeiss LSM510 confocal microscope at 40X magnification. Images were captured using the tile function in Volocity, V5.5.1 (PerkinElmer, Waltham, MA, USA).

### Nucleic acid extraction

For in vitro cultures 300 μl lysis buffer (Norgen Biotek, Thorold, ON, Canada) was added to each well and cells were harvested by scraping. Ocular swabs were thawed and added to a 1.5 ml microcentrifuge tube containing 300 μl lysis buffer (Norgen Biotek) and were vortexed for one minute. Total RNA and DNA were extracted from both sample types using the Norgen RNA and DNA purification kit according to the manufacturers instructions. Total RNA was quantified using Qubit® fluorometric quantitation (Life Technologies) and quality was assessed using an Agilent RNA 6000 pico kit on a 2100 Bioanalyzer instrument (Agilent technologies, Santa Clara, CA, USA).

### Ct infection load by droplet digital PCR

A droplet-digital PCR assay was carried out to diagnose Ct infection, as described previously [25, 26] with the following modifications. Forward and reverse primers were used at 0.9 μM and probes were used 0.2 μM. Eight microlitres of sample DNA was added to each reaction. For clinical samples, an initial diagnostic assay detecting Ct plasmid and human RPP30 endogenous control was carried out on all samples. Ct *omcB* and plasmid DNA was then quantified in positive samples to determine Ct load. This assay was also performed on in vitro samples to quantitate Ct *omcB* load and to check that there was no cross contamination of plasmid competent Ct in plasmid-free Ct cultures. Primer and probe sequences are described elsewhere [26]. Thermal cycling conditions were as follows: 95 °C hold for 10 min, followed by 40 cycles of 95 °C for 10 s and 60 °C for 20 s with a final hold of 98 °C for 12 min. ddPCR was performed on a Bio-Rad QX100 Instrument and data were collected using Quantalife software (Bio-Rad, Hercules, CA, USA). Copy number was calculated in R using previously published scripts [25].

### Small RNA sequencing

Small RNA libraries were prepared using TruSeq® Small RNA sample preparation kit (Illumina, San Diego, CA, USA), carried out following the manufacturer’s instructions. In vitro-derived samples chosen for sequencing had an RNA Integrity number (RIN) > 8 and a concentration of at least 0.2 μg/μl total RNA. Sample concentrations were normalized prior to small RNA library preparation. Clinical samples chosen for sequencing had an RIN > 8 and were normalized to the sample with the lowest concentration (24 ng/μl). Libraries were sequenced on a MiSeq desktop sequencer (Illumina). Sequencing reads were analyzed as described previously [27] with an amendment such that only reads with a Phred33 score of 30 or more were retained. Differential analysis was performed on read count data using two analysis packages in R, DESeq (which is believed to be conservative) and EdgeR [28–30]. In both methods miR with an average read count across all samples of less than five were excluded from each analysis. *P* values were adjusted for false discovery rate (FDR) using the Benjamini-Hochberg procedure [31]. Raw and processed sequencing data along with processing workflows are deposited in the NCBI GEO public database (GSE69837).

### MiR qPCR and statistical analysis

Total RNA was reverse transcribed and expression levels were quantified by quantitative PCR (qPCR) using the MiScript system (Qiagen, Venlo, Limburg, Netherlands) as described previously [15]. Thermal cycling conditions were 95 °C for 15 min followed by 40 cycles of 94 °C for 15 s, 55 °C 30 s, and 70 °C 30 s. Data was collected at 70 °C. Cycle thresholds were calculated using an automatic baseline and a threshold of 0.1 for all clinical and in vitro samples. Differential expression analysis of cycle threshold values was carried out as described previously [15]. FDR adjusted *P* values were calculated using the Benjamini-Hochberg procedure.

The relationship between conjunctival papillary inflammation (P) score and the expression of miR that were significantly differentially expressed in TF versus N by qPCR was examined using ordinal logistic regression models in R (MASS package [32]). Clinical scores P2 and P3 were combined due to the low number of P3 scores (*n* = 6) and individuals were classified as P0 (*n* = 84), P1 (*n* = 53) and P2/P3 (*n* = 29) as an ordinal response variable. Age, gender and the natural logarithm of Ct *omcB* load were included in the model and miR expression levels (40-delta cycle threshold (∆CT) value) were included as independent variables. This value (40-∆CT) was used so that an odds ratio (OR) >1 would reflect an up-regulation of miR expression and vice versa. Univariable ordinal regression was performed for each miR and miR with a *P* value < 0.05 were included in the final multivariate model. F and P scores were tested for collinearity: A high kappa score and a covariance matrix value near zero indicates collinearity (0 = perfect colinearity, 1 = no colinearity). Post-hoc analyses were carried out on miR with a *P* value <0.05 in the multivariate model. Kruskal-Wallis and post-hoc Kruskal Nemenyi tests (Tukey method) were carried out in R using the PMCMR package [33] to detect pairwise differences between miR expression and collapsed papillary inflammation score. Kruskal Nemenyi test *P* values were adjusted using the Benjamini-Hochberg procedure.

## Results

### In vitro infections

**Fig. 1:**
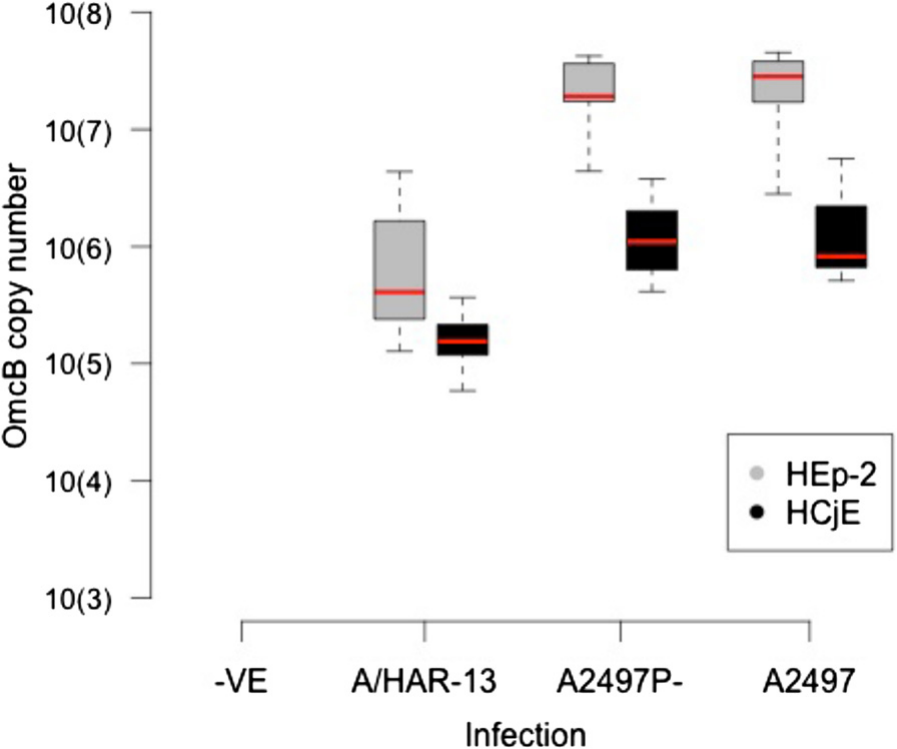
Yield of three ocular Ct strains in two epithelial cell lines. Chlamydial genome copy number of three ocular Ct strains (A2497, A2497p- and A/HAR-13) in HEp-2 and HCjE cells at 48 hpi. Cells were infected at an MOI of 1. Mock-infected cells (−VE) were treated identically except for the addition of Ct inoculum. The mean of the total OmcB genome copy number per well of 6 biological replicates is shown

HEp-2 and HCjE epithelial cell lines were infected in vitro with 3 different serovar A ocular strains of Ct; A/HAR-13, A2497 and plasmid-cured A2497. In HEp-2 cells, virulent A2497 plasmid-competent Ct had the highest mean genome copy number at 48 hpi, which was slightly higher than that of isogenic plasmid-free A2497 (A2497P-) (Fig. 1). A/HAR-13 growth was considerably less efficient in these experiments. All 3 strains were substantially less productive in HCjE cells compared to HEp-2 cells, as measured by *omcB* load and microscopy of inclusions (Fig. 1 and Additional file 1). Due to the low productivity of A/HAR-13 infection in HEp-2 and HCjE cells, these samples were not carried forward for small RNA sequencing. A2497 and A2497P- infected HCjE cells were sequenced despite the poor infection productivity in order to contrast small RNA expression between infected HEp-2 and HCjE cells. Small RNA sequencing was performed on mock-infected, A2497 and A2497P- infected HEp-2 and HCjE cells.

In HEp-2 cells, 885 miR were detected in mock-infected cells, 894 miR were detected in A2497 infected cells and 856 miR were detected in A2497P- infected cells. Differential expression of read count data (miR with an average read count <5 excluded) was carried out using EdgeR and DEseq to account for variance in the normalization method. No miR were differentially expressed (P_adj_ <0.01) using either method between mock-infected and A2497 infected (377 miR tested) or between mock-infected and A2497P- infected (378 miR tested) HEp-2 cells (Additional file 2). In HCjE cells, 853 miR were detected in mock-infected cells, 840 miR were detected in A2497 infected cells and 872 miR were detected in A2497P- infected cells. No miR were differentially expressed using EdgeR or DESeq (P_adj_ <0.01) between mock-infected and A2497 infected (357 miR tested) or between mock-infected and A2497P- infected (372 miR tested) HCjE cells (Additional file 3).

The miR profile of mock-infected HEp-2 and HCjE cells was analyzed in order to investigate differences between these two cell lines. Four hundred and seven miR had an average read count > 5 in mock-infected HEp-2 and HCjE cells. Strikingly, DESeq identified 136 out of the 407 miR tested with an adjusted *P* value (P_adj_) < 0.01 and EdgeR identified 217 miR with P_adj_ < 0.01 (Additional file 4). The top 3 most differentially expressed miR were members of the miR-200 family: miR-200b-3p (P_adj_ = 2.11 × 10^−51^, log_2_FC = 12.3), miR-141-5p (P_adj_ = 1.1 × 10^−32^, log_2_FC = 9.2), and miR-429 (P_adj_ = 1.1 × 10^−32^, log_2_FC = ∞), which were all highly expressed in HCjE cells relative to HEp-2 cells. MiR-429 was detected at an average read count of 379 in mock-infected HCjE cells, whereas it was not detected at all in HEp-2 cells at the sequencing depth achieved.

### Clinical sample details

Conjunctival swab samples were collected from 81 cases of TF and 82 clinically normal healthy controls. A summary of sample phenotypes is shown in Table 1. Five out of 82 controls and 47/81 cases were positive for Ct DNA (Chi^2^ = 55.93, *P* = 0.0005). The proportion of Ct infected individuals in each clinical category increases with the severity of trachomatous inflammation, both follicular and papillary. Two clinical cases had evidence of conjunctival scarring; one sample was F2, P1, C1 (Ct positive) and the other was F3, P2, C2 (Ct negative). The median Ct plasmid copy number was 1.5 (range 0.4–14.6).

**Table 1:** Trachoma grade and Ct infection load of clinical samples

FPC score	Total (% male)	Ct positive	Ct *omcB* median^a^ copies/swab (IQR)^b^	Human RPP30 median copies/swab (IQR)^b^
Controls (N)				
F0P0C0	82 (41 %)	5/82 (6 %)	345 (42–1078)	19290 (13310–25810)
TF cases: Breakdown by follicular score				
F2	46 (48 %)	20/46 (43.5 %)	703 (370–25870)	23900 (15670–39440)
F3	35 (31 %)	27/35 (77 %)	6050 (1451–15350)	35250 (19120–43250)
TF cases: Breakdown by papillary hypertrophy score				
P1	53 (38 %)	22/53 (71 %)	656 (433–5238)	23280 (15380–34500)
P2	22 (55 %)	19/22 (86 %)	12950 (3638–42880)	37750 (21560–48560)
P3	6 (17 %)	6/6 (100 %	11480 (4169–15720)	43620 (37750–53440)

^a^Ct omcB median calculated from Ct positive individuals only

^b^IQR = inter-quartile range

Fifty-one of the 163 clinical samples from which total RNA was extracted had an RIN > 8. Five of the 51 RIN > 8 samples with the highest RNA concentration that had TF with current Ct infection were chosen for sequencing. Five control samples (F0,P0,C0) with the highest RNA concentration and the closest age and sex match to the 5 TF samples were also sequenced. Total RNA concentration of all 10 samples was normalized to the sample with the lowest concentration (24 ng/μl) prior to library preparation. The mean age of the 5 controls was 6.4 years old and the group consisted of 2 males and 3 females. The mean age of the TF group was 6.2 years old and the group consisted of 3 males and 2 females. In the TF group, the sample phenotypes were F2,P1,C0 (*n* = 2), F2,P2,C0 (*n* = 1), F3,P1,C0 (*n* = 1) and F3,P2,C0 (*n* = 1).

### MiR expression in clinical samples by RNAseq

Deep sequencing of conjunctival small RNA from 10 clinical samples identified 860 miR. MiR-21-5p was the most highly expressed miR in the conjunctiva of both N and TF cases (Additional file 5). MiR with an average read count < 5 in all ten samples were removed. EdgeR revealed 43 miR (P_adj_ < 0.01, FC > 1.5) and DEseq identified 12 miR (P_adj_ < 0.01 FC > 1.5 (Additional file 6)) that were differentially expressed between TF cases and controls. All of the significant miR identified by DEseq were also significant using EdgeR.

**Fig. 2:**
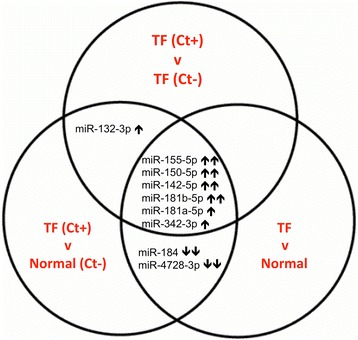
Patterns of differential miR expression according to clinical phenotype. MiR expression was measured using qPCR in 163 clinical samples. MiR labeled with double arrows had significant differences between groups (P_adj_ < 0.05, FC > 1.5). MiR with single arrows had significantly different expression (P_adj_ < 0.05) but had a FC <1.5 in 2 or 3 of the comparisons shown

The differential expression of the 12 miR that were significant in both DESeq and EdgeR analyses was investigated in 163 clinical samples using qPCR. Two miR that were previously found to be up-regulated in inflammatory trachomatous scarring (miR-147b and miR-1285) [15] were also tested by qPCR in these samples. Results were analyzed using the ΔΔCt method [34]. Differential expression was tested in 3 independent clinical phenotype comparisons: TF (*n* = 81) against normal healthy controls (N, *n* = 82), TF with detectable Ct infection (TF Ct + *n* = 47) against normal healthy controls without detectable Ct infection (N Ct-, *n* = 77), and TF with detectable Ct infection (TF Ct+, *n* = 47) against TF cases without detectable Ct infection (TF Ct-, *n* = 34). Results are shown in Table 2. MiR-155, miR-150, miR-142, miR-181b, miR-181a and miR-342 were up-regulated in all 3 comparisons (Fig. 2). MiR-155, miR-150, miR-142, miR-181b, miR-181a, miR-342 and miR-132 were differentially expressed during current Ct infection. MiR-184 and miR-4728 were down-regulated in TF independently of Ct infection. MiR-184 was the only miR that was significantly differentially expressed between uninfected normal healthy controls (*n* = 77) and Ct negative TF cases (TF Ct- (*n* = 34), P_adj_ = 0.00165, FC = 0.315, data not shown). These results are consistent with the small RNA sequencing results (Additional file 6). MiR-147b and miR-1285, which were up-regulated in inflammatory trachomatous scarring [15], were not differentially expressed between TF/TI and N.

**Table 2:** Differential expression analysis of miR by qPCR in 163 clinical samples, in three independent phenotype comparisons

	TF (*n* = 81) v N (*n* = 82)			TF Ct + (*n* = 47) V TF Ct- (*n* = 34)			TF Ct + (*n* = 47) V N Ct- (*n* = 77)		
	*P* value	Adj P^a^	FC^b^	*P* value	Adj P^a^	FC^b^	*P* value	Adj P^a^	FC^b^
miR-184	5.69 ^x^ 10^−8^	7.96 ^x^ 10^−7^	0.30	0.7571	0.7571	0.88	3.06 ^x^ 10^−7^	1.07 ^x^ 10^−6^	0.28
miR-142-5p	6.46 ^x^ 10^−7^	4.52 ^x^ 10^−6^	1.82	1.63 ^x^ 10^−5^	7.58 ^x^ 10^−5^	2.53	2.3 ^x^ 10^−12^	3.22 ^x^ 10^−11^	2.83
miR-155-5p	1.56 ^x^ 10^−5^	7.29 ^x^ 10^−5^	1.73	9.45 ^x^ 10^−6^	6.62 ^x^ 10^−5^	2.50	1.18 ^x^ 10^−9^	5.51 ^x^ 10^−9^	2.58
miR-150-5p	0.0003	0.0011	1.56	1.41 ^x^ 10^−6^	1.98 ^x^ 10^−5^	3.24	8.56 ^x^ 10^−10^	5.51 ^x^ 10^−9^	2.71
miR-181b-5p	0.0010	0.0028	1.33	0.0018	0.005	1.74	1.58 ^x^ 10^−6^	4.44 ^x^ 10^−6^	1.70
miR-342-3p	0.0041	0.0096	1.12	0.0076	0.0178	1.50	0.0001	0.0002	1.34
miR-4728-3p	0.0103	0.0207	0.75	0.0353	0.0617	0.72	0.0005	0.0008	0.65
miR-181a-5p	0.0120	0.0211	1.30	0.0151	0.0301	1.40	0.0003	0.0006	1.54
miR-375	0.0871	0.1355	0.80	0.3054	0.3887	0.81	0.0615	0.0862	0.75
miR-132-3p	0.1628	0.2279	1.09	0.0009	0.0031	1.45	0.0023	0.0035	1.27
miR-10a-5p	0.2654	0.3378	1.16	0.6729	0.7247	0.92	0.3454	0.4030	1.15
miR-146b-3p	0.5491	0.6406	0.95	0.3666	0.4277	1.15	0.9425	0.9425	1.02
miR-147b	0.6203	0.6680	1.07	0.1073	0.1669	1.44	0.2394	0.3047	1.21
miR-1285	0.8683	0.8683	0.98	0.2095	0.2933	0.83	0.4434	0.4775	0.91

Tests for difference were conducted with a *T* test or Wilcoxon signed rank test if data were not normally distributed

*N* normal, *TF* follicular trachoma, *Ct +* Ct infected, *Ct-* no Ct infection detected

^a^P adj = Adjusted *P* value

^b^FC = Fold change

### Relationship between clinical papillary inflammation and miR expression

**Fig. 3:**
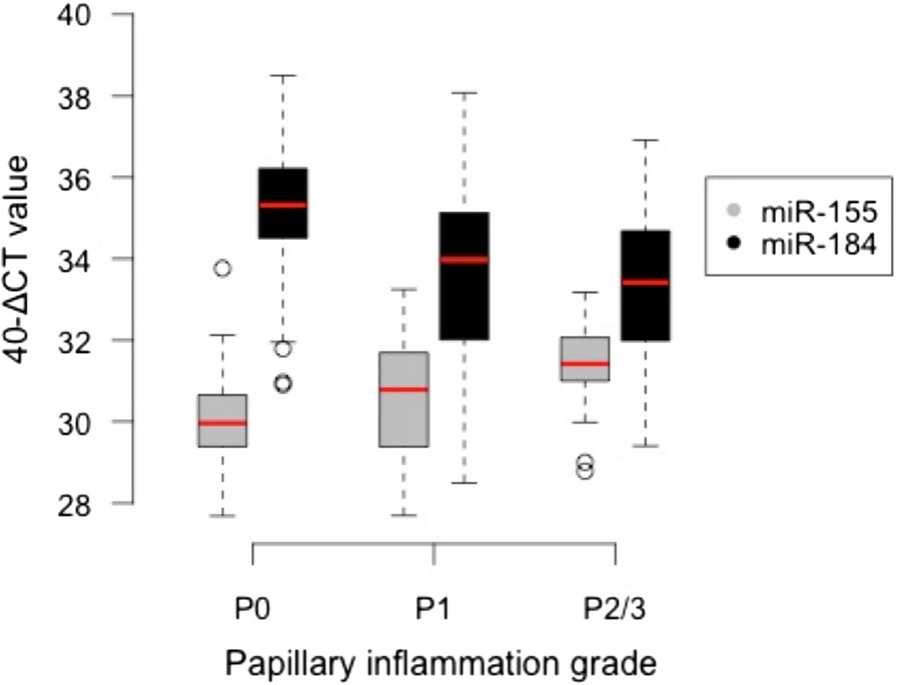
MiR expression correlates with clinical inflammation score. Expression of miR-155 and miR-184 for 163 clinical samples plotted against clinical trachoma papillary hypertrophy score. P2 and P3 categories were combined due to the low number of P3 cases. ∆CT values were calculated by subtracting the endogenous control (snoRNA U6) cycle threshold (CT) value from the CT value of each miR for each individual sample. ∆CT values were inverted (40-∆CT) in order to show the direction of miR expression

We investigated the relationship between clinical papillary inflammation and miR expression using an ordinal logistic regression model, which allowed us to adjust for confounding variables and estimate the contribution of miR expression to inflammation. Ct load was adjusted for in order to detect miR associated with papillary inflammation independently of Ct infection. Follicular trachoma and papillary hypertrophy were highly collinear (Kappa score = 5.8^ x ^10^15^, determinant of covariance matrix = 1.92 ^x^ 10^−21^) therefore F score was not included as an independent variable in this analysis as the purpose of this model was to investigate papillary inflammation. MiR-150, −142, −4728, −181A and -181B were up-regulated in follicular trachoma in response to current Ct infection (Fig. 2) and were associated with papillary inflammation in univariate regression analyses, but they had no association with papillary inflammation after adjustment for Ct infection load (Additional file 7). MiR-155 (OR = 2.533 (95 % CI = 1.291–4.971), *P* = 0.007) and miR-184 (OR = 0.416 (95 % CI = 0.300–0.578), *P* = 1.61 ^x^ 10^−7^) were significantly associated with papillary inflammation (Additional file 7). MiR-155 expression increases with papillary inflammation score (Fig. 3). Significant differences in miR-155 expression were found between P0 and P1 (*P*_*adj*_ = 0.03), P1 and P2/3 (*P*_*adj*_ = 0.019) and P0 and P2/3 (*P*_*adj*_ = 3.6 ^x^ 10^−6^). MiR-184 expression decreased with papillary inflammation score (Fig. 3). Significant differences in miR-184 expression level were found between P0 and P1 (*P*_*adj*_ = 5.5 ^x^ 10^−5^) and between P0 and P2/3 (*P*_*adj*_ = 4.2 ^x^ 10^−5^). In separate univariate multinomial logistic regression analyses, no associations were found between miR expression and plasmid copy number in Ct positive individuals (data not shown). Exclusion of the two samples with evidence of trachomatous scarring did not change the outcomes of the differential expression or regression analyses (data not shown).

## Discussion

During Ct infection of the conjunctiva in vivo 7 miR were up-regulated. Two miR were down-regulated during follicular trachoma in the absence of Ct. After adjusting for the relationship between miR and Ct infectious load, we found that miR-155 and miR-184 were independently associated with inflammation. We also found that isolated epithelial cells in culture did not differentially regulate miR expression in response to Ct infection.

The Ct plasmid is a known virulence factor [35, 36]. In a non-human primate model of trachoma A2497P- infection is attenuated relative to A2497 and does not lead to ocular pathology [24]. Attempts to elucidate the mechanism of this attenuation are ongoing [37, 38]. In order to understand the contribution of the epithelium, Porcella et al. (2015) recently characterized the in vitro transcriptome response of HeLa cells infected with A2497 and A2497P- (at an MOI of 1) [39]. Similar transcriptional profiles were induced by A2497 and A2497P- and only modest increases in cytokine and chemokine levels were detected in response to virulent A2497. The authors suggested that their data supports the hypothesis that Ct is a stealth pathogen [40], due to the limited innate pro-inflammatory response induced. We did not detect differential regulation of miR by deep sequencing in HEp-2 or HCjE epithelial cells at 48 hpi with A2497 and A2497P- (also at an MOI of 1). This result is not surprising in HCjE cells due to the lack of productive infection achieved, however more differences might have been expected upon productive infection of HEp-2 cells, particularly as Porcella et al., found the largest transcriptional changes occurred in response to A2497 and A2497P- at 48hpi [39]. Our data could also be interpreted as evidence in support of the stealth pathogen hypothesis and suggests that Ct avoids stimulation of a miR response in epithelial cells. Infection of HeLa cells with *Salmonella typhimurium* revealed the largest changes in miR expression at 4hpi, with minimal changes detected at 24hpi despite infecting with an MOI of 10 [41]. Therefore, it is also possible that changes in epithelial cell miR expression in response to Ct are limited to early time points post infection. Limitations of this experiment were that despite infecting with an MOI of 1 we achieved <50 % infectivity (Additional file 1) and we sequenced three biological repeats per condition. The expression changes in infected cells were therefore likely diluted by the lack of expression change in neighboring uninfected cells and it is possible that we did not have the statistical power to detect small expression differences.

HEp-2 cells originated from a human laryngeal carcinoma but it is known that many laboratory stocks of HEp-2 cells are contaminated with HeLa cells, which are derived from a glandular adenocarcinoma of the cervix [42]. HCjE cells are an immortalized cell line generated from a primary conjunctival epithelial culture [43] and may represent an in vitro model that better mimics conjunctival epithelial cells in vivo. A previous study infected HCjE and HeLa cells with A/HAR-13 at an MOI of 0.3 and reported that A/HAR-13 achieved higher infectious load in HCjE cells compared to HeLa cells [44]. In contrast we were unable to maintain productive infections of 3 ocular strains in HCjE cells, whereas productive infection of HEp-2 cells with A2497 and A2497P- was achieved. These differences could reflect culture adaptation of HCjE cells [45–48] or Ct strains [49] over time or a natural resistance of the HCjE line to infection, perhaps due to expression of mucins or innate antimicrobial effectors [50]. Comparison of mock-infected HEp-2 and HCjE cell lines revealed significant differences in miR profile. Amongst the miR that were most significantly up-regulated in HCjE cells relative to HEp-2 cells were several members of the miR-200 family (Additional file 4), which are known to be highly enriched in epithelial tissues [51]. This would suggest that HEp-2 cells present an environment to Ct that is different to conjunctival epithelial cells and might indicate that caution should be used when interpreting data generated from these cultures in comparison to the in vivo response.

In vivo, we found that miR-21-5p was the most abundant miR in the conjunctiva of both follicular trachoma cases and healthy control individuals (Additional file 5). MiR-21 is transactivated by NFκB [52] and is thought to act in a positive feedback loop in epithelial cells by increasing NFκB activity [53]. The high expression of miR-21 at this mucosal site may reflect NFκB stimulation in an environment of frequent microbial exposure. We previously found that miR-21 was abundant (Ranked 19/503) in the conjunctiva of Gambian adults with and without scarring trachoma [15]. This slight reduction in the relative abundance of miR-21 in adults may reflect the fact that the conjunctival microbiome of adults in The Gambia has reduced richness and diversity of species compared to Gambian children [54] and thus a lower level of microbial stimulation. The reduction in abundance could also be due to differences in microbial exposure between The Gambia and Guinea Bissau, or the different technical approaches used in the two studies such as small RNA purification and quantitation.

In follicular trachoma, the miR expression signature reflects the presence of the immune response. MiR-155, miR-150 and miR-142 are thought to be specific for hematopoietic cells [55] (although miR-155 expression has been reported in epithelial cell lines [56, 57]) and miR-155, miR-150, miR-181a, miR-146 and miR-10a have roles in hematopoiesis (reviewed in [58]). MiR-155 in particular has wide-ranging and profound effects on the development and function of immune cells (reviewed in [59]). MiR-181b, miR-132, miR-10a and miR-146b negatively regulate inflammation following TLR or NFκB stimulation in order to prevent excessive inflammation and pathology. A brief summary of roles that have been described for these differentially regulated miR relating to inflammation or fibrosis is presented in Additional file 8. The expression of these miR following stimulation of innate pathogen recognition pathways explains their up-regulation in response to Ct in this study. We found that expression of miR-150, −181a, −181b, −142 and −4728 were each independently associated with increasing inflammatory score, however, these associations were no longer significant after adjustment for Ct load and miR-co-expression. MiR-155 and miR-184 alone had a strong and direct association with papillary inflammatory score after adjustment for these factors. Pathway analysis was not carried out due to the relatively small and manageable number of differentially expressed miR and the inherent bias of current miR-based pathway analysis software [60].

A number of the miR that were differentially regulated in Ct positive follicular trachoma cases have previously been linked to chlamydial disease. MiR-155-5p, miR-142-5p, miR-142-3p, miR-132-3p and miR-147-3p amongst others were up-regulated during Cm infection of the murine cervix in response to an attenuated strain of Cm, relative to a virulent strain of Cm [16]. The roles attributed to these miR in dampening inflammation possibly explain the reduced pathology observed upon avirulent infection. Another study found that miR-146a was up-regulated in the murine genital tract 6 days post infection with Cm [17]. We did not find miR-147b or miR-1285 to be differentially expressed in Ct positive or Ct negative follicular trachoma cases. This could reflect differences in the inflammatory phenotype between adults with trachomatous scarring and inflammation (TI) that continues in the absence of Ct, compared to younger individuals with chlamydial-induced inflammation (TF and TI). MiR-147b is also hypothesized to dampen inflammation upon LPS and TNFα stimulation [61, 62], therefore regulation of inflammation is a key theme in both stages of trachomatous disease. No differentially regulated miR were detected in healthy control adults versus individuals with scarring trachoma in our previous study; differences were only detected in comparisons against trachomatous scarring with inflammation [15]. These data, combined with recent evidence that inflammation is a significant risk factor for progressive scarring [1], support the hypothesis that inflammatory cells are required to drive pathological responses in the epithelium. This hypothesis perhaps also explains the lack of miR response in our infected epithelial cell model and suggests that inflammatory cell stimuli are required for a miR response in the epithelium in vivo, as might be the case for miR-184.

We found that miR-184 and miR-4728-3p were down-regulated in follicular trachoma irrespective of Ct infection. MiR-184 was the only miR differentially expressed in uninfected cases of follicular trachoma versus uninfected healthy controls and was highly associated with papillary hypertrophy score after adjustment for Ct load. MiR-184 is known to be enriched in the corneal epithelium [63] and it is the fourth most abundant miR in control individuals (F0P0C0). We previously found that miR-184 was highly abundant (5/502) in a pool of normal healthy adults and adults with scarring trachoma [15], suggesting tissue specific expression does not vary with age. A single base substitution in the seed region of miR-184 is associated with severe keratoconus (thinning of the cornea) [64–66]. In the cornea miR-184 is expressed in basal and supra-basal epithelial cells [63], under which the stromal thinning occurs. MiR-184 is strongly down-regulated during acute corneal injury and expression is restored in the re-epithelialized cornea upon healing [63]. MiR-184 also targets the Wnt receptor frizzled-7 and negatively regulates the Wnt pathway; down-regulation of miR-184 is associated with aberrant activation of the Wnt pathway in ischemia-induced neovascularization of the murine retina [67]. Delivery of miR-184 was able to inhibit induction of the Wnt pathway, conferring miR-184 with significant therapeutic potential. Activation of the canonical Wnt pathway has previously been demonstrated in Ct infected epithelial cells in ex-vivo fallopian tube tissue [68], with a paracrine effect that resulted in a loss of epithelial homeostasis. Altogether, these data suggest that a down-regulation of miR-184 in follicular trachoma once Ct has been cleared reflects a prolonged wound healing process and activation of the Wnt pathway, possibly contributing to pathology. Prolonged down-regulation of miR-184 may also contribute to epithelial thinning, which is observed in trachoma, potentially predisposing individuals more to subsequent bacterial infections. Future work should focus on the expression of miR-184 and activity of the Wnt pathway in a longitudinal set of clinical samples to determine the contribution of this pathway to the development of trachomatous pathology.

There was considerable variation in load between the Ct infected follicular trachoma cases in this study. Conjunctival cells obtained by a swab are expected to be heterogeneous in the number of infected and uninfected epithelial cells and the number of immune cells. The majority of epithelial cells are likely to be uninfected; reducing our power to detect miR expression differences in infected epithelial cells and perhaps explaining why the majority of differentially regulated miR detected in vivo were hematopoietic cell-associated. This heterogeneity is a caveat of using clinical samples. In order to investigate the host miR response of Ct infected epithelial cells in vivo infected cells could be isolated and subjected to single cell miRNA expression analysis [69].

## Conclusions

We identified 7 miR that were up-regulated by current chlamydial infection during follicular trachoma. MiR-184 and miR-4728 were down-regulated during follicular trachoma in the absence of Ct. MiR-155 and miR-184 were inversely correlated with pathological inflammation, which is known to be a major risk factor for scarring trachoma. Future studies using more sophisticated in vitro models (such as co-culture) involving miR silencing or overexpression coupled with longitudinal studies in vivo will help develop our understanding of the functions of these miR, their use as predictive biomarkers of scarring trachoma and their potential as novel treatment targets.

## Additional files

Additional file 1: **Chlamydial infection of HEp-2 and HCjE cell lines with three ocular Ct strains.** HEp-2 cell monolayers infected with Ct strains A2497 (A), A2497P- (B) and A/HAR-13 (C), and HCjE cell monolayers infected with Ct strains A2497 (D), A2497P- (E) and A/HAR-13 (F) at 48 hpi. Cell nuclei are stained with DAPI (blue) and Ct inclusions are stained with anti-C. trachomatis MOMP antibody (FITC (green)). Cells were infected with an MOI of 1 and viewed at 40X magnification. Representative photographs were taken from one of three replicates for each condition. (PDF 770 kb)

Additional file 2: **Differential expression analysis of miR from A2497 and A2497P- infected HEp-2 cells.** Results of DESeq differential expression analysis of sequencing read count data for uninfected HEp-2 cells versus HEp-2 cells infected with A2497 and A2497P- strains of Chlamydia trachomatis at 48hpi. MiR with an average read count <5 were excluded. Three biological replicates of each condition were sequenced and analyzed. MRC = Mean read count, −VE = mock-infected cells. (XLS 141 kb)

Additional file 3: **Differential expression analysis of miR from A2497 and A2497P- infected HCjE cells.** Results of DESeq differential expression analysis of sequencing read count data for uninfected HCjE cells versus HCjE cells infected with A2497 and A2497P- strains of Chlamydia trachomatis at 48hpi. MiR with an average read count <5 were excluded. Three biological replicates of each condition were sequenced and analyzed. MRC = Mean read count, −VE = mock-infected cells. (XLS 137 kb)

Additional file 4: **Differential expression analysis of miR from uninfected HCjE versus HEp-2 cells.** Results of DESeq differential expression analysis of sequencing read count data for uninfected HEp-2 cells versus uninfected HCjE cells at 48hpi. MiR with an average read count <5 were excluded. Three biological replicates of each condition were sequenced and analyzed. MRC = Mean read count, −VE = mock-infected cells. (XLS 102 kb)

Additional file 5: **Relative abundance of miR in the conjunctiva of cases of follicular trachoma (TF) and controls (N).** MiR with a mean read count in TF cases or controls < 5 were excluded. MiR were ranked by mean read count across all five samples of each phenotype group. (PDF 678 kb)

Additional file 6: **Differential expression analysis of miR from individuals with follicular trachoma versus healthy controls.** Results of DESeq differential expression analysis of sequencing read count data for normal healthy controls (N) versus cases of follicular trachoma (TF) with current Chlamydia trachomatis (Ct) infection (five samples of each). MiR with an average read count <5 were excluded. MRC = Mean read count. (XLS 73 kb)

Additional file 7: **Multivariable regression model of the contribution of miR expression to clinical papillary hypertrophy score.** Collapsed papillary hypertrophy score (P0, P1 or P2/3, as defined by the WHO 1981 FPC scoring system) was used as an ordinal outcome variable to define trachomatous inflammation in 163 clinical samples. Age, gender, Ct load and inverted ∆CT values (40-∆CT) of miR are included as independent variables. Model AIC (Akaike information criterion) is 227.3725. ^a^OR = Odds ratio, ^b^CI = confidence intervals, ^c^Ct load is defined as log-(e) omcB copies/swab. (PDF 59 kb)

Additional file 8: **Focused list of published roles for miR that are differentially expressed in follicular trachoma that relate to inflammation or fibrosis.** (PDF 59 kb)

## Abbreviations

∆CT: delta cycle threshold value
CI: confidence interval
Ct: Chlamydia trachomatis
F score: follicle score
hpi: hours post-infection
miR: microRNA
MOI: multiplicity of infection
N: normal healthy control
OR: odds ratio
P score: papillary inflammation score
qPCR: quantitative polymerase chain reaction
RIN: RNA integrity number
TF: trachomatous inflammation – follicular
TI: trachomatous inflammation – intense
TLR: toll-like receptor
