# Supplementary material for: Inverse relationship between microRNA-155 and -184 expression with increasing conjunctival inflammation during ocular Chlamydia trachomatis infection
Source: BMC Infect Dis. 2016 Feb 3;16:60. doi: 10.1186/s12879-016-1367-8 (PMC4739388; doi:10.1186/s12879-016-1367-8)
Supplement: Supplementary file 1 — Chlamydial infection of HEp-2 and HCjE cell lines with three ocular Ct strains. HEp-2 cell monolayers infected with Ct strains A2497 (A), A2497P- (B) and A/HAR-13 (C), and HCjE cell monolayers infected with Ct strains A2497 (D), A2497P- (E) and A/HAR-13 (F) at 48 hpi. Cell nuclei are stained with DAPI (blue) and Ct inclusions are stained with anti-C. trachomatis MOMP antibody (FITC (green)). Cells were infected with an MOI of 1 and viewed at 40X magnification. Representative photographs were taken from one of three replicates for each condition. (PDF 770 kb) [file 12879_2016_1367_MOESM1_ESM.pdf]

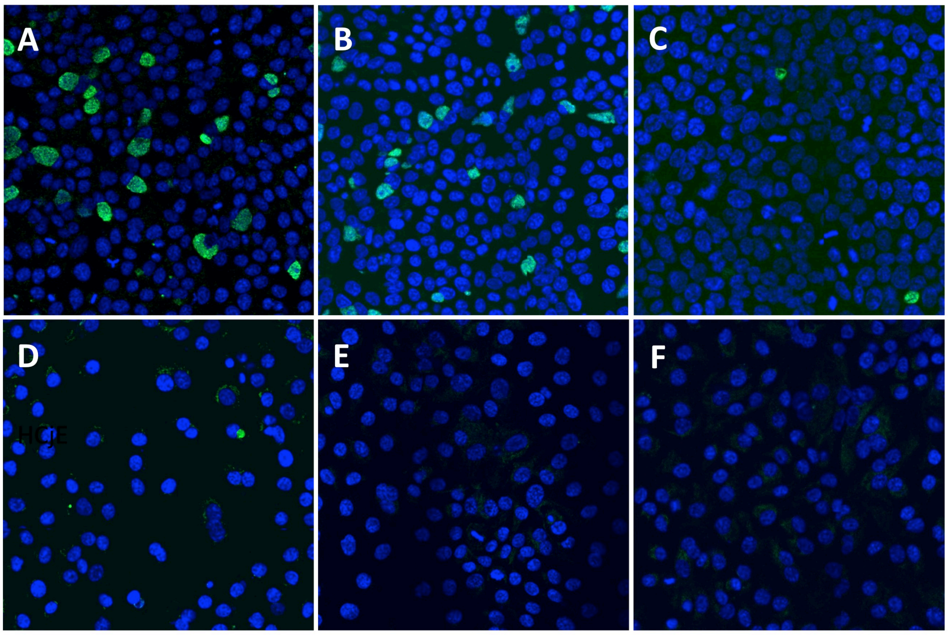

*Additional file 1. HEp-2 cell monolayers infected with Ct strains A2497 (A), A2497P- (B) and A/HAR-13 (C), and HCjE cell monolayers infected with Ct strains A2497 (D), A2497P- (E) and A/HAR-13 (F) at 48 hpi. Cell nuclei are stained with DAPI (blue) and Ct inclusions are stained with anti-*C. trachomatis *MOMP antibody (FITC (green)). Cells were infected with an MOI of 1 and viewed at 40X magnification. Representative photographs were taken from one of three replicates for each condition.*
