# Supplementary material for: Inverse relationship between microRNA-155 and -184 expression with increasing conjunctival inflammation during ocular Chlamydia trachomatis infection
Source: BMC Infect Dis. 2016 Feb 3;16:60. doi: 10.1186/s12879-016-1367-8 (PMC4739388; doi:10.1186/s12879-016-1367-8)
Supplement: Supplementary file 5 — Relative abundance of miR in the conjunctiva of cases of follicular trachoma (TF) and controls (N). MiR with a mean read count in TF cases or controls < 5 were excluded. MiR were ranked by mean read count across all five samples of each phenotype group. (PDF 678 kb) [file 12879_2016_1367_MOESM5_ESM.pdf]

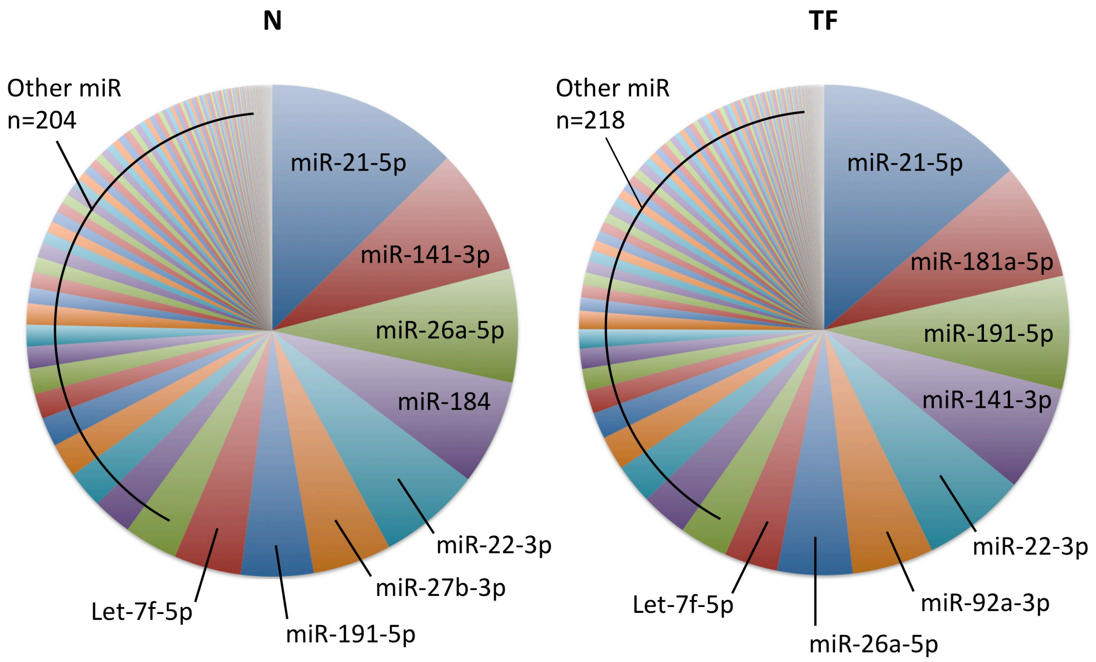

Additional file 5. Abundance of miR in the conjunctiva of cases of TF and controls (N). miR with a mean read count in cases or controls < 5 were excluded. MiR were ranked by mean read count across all five samples of each phenotype group.
