# Supplementary material for: Inverse relationship between microRNA-155 and -184 expression with increasing conjunctival inflammation during ocular Chlamydia trachomatis infection
Source: BMC Infect Dis. 2016 Feb 3;16:60. doi: 10.1186/s12879-016-1367-8 (PMC4739388; doi:10.1186/s12879-016-1367-8)
Supplement: Supplementary file 7 — Multivariable regression model of the contribution of miR expression to clinical papillary hypertrophy score. Collapsed papillary hypertrophy score (P0, P1 or P2/3, as defined by the WHO 1981 FPC scoring system) was used as an ordinal outcome variable to define trachomatous inflammation in 163 clinical samples. Age, gender, Ct load and inverted ∆CT values (40-∆CT) of miR are included as independent variables. Model AIC (Akaike information criterion) is 227.3725. aOR = Odds ratio, bCI = confidence intervals, cCt load is defined as log-(e) omcB copies/swab. (PDF 59 kb) [file 12879_2016_1367_MOESM7_ESM.pdf]

Additional file 7. Multivariable regression model of the contribution of miR expression to clinical papillary hypertrophy score.

**Independent variable**	**Adjusted OR[a]**	**95% CI[b]**	**P value**
Age (years)	1.247	1.034 – 1.503	0.0210
Gender (Male)	1.145	0.536 – 2.446	0.7266
Ct load[c]	1.256	1.053 – 1.498	0.0113
miR-155	2.533	1.291 – 4.971	0.0069
miR-184	0.416	0.300 – 0.578	$1.61*10^{-7}$
miR-150	0.654	0.349 – 1.227	0.1861
miR-181a	1.195	0.600 – 2.382	0.6118
miR-181b	1.623	0.779 – 3.381	0.1962
miR-142	1.369	0.794 – 2.357	0.2582
miR-4728	0.718	0.479 – 1.076	0.1084

Collapsed papillary hypertrophy score (P0, P1 or P2/3, as defined by the WHO 1981 FPC scoring system) was used as an ordinal outcome variable to define trachomatous inflammation in 163 clinical samples. Age, gender, Ct load and inverted ΔCT values (40-ΔCT) of miR are included as independent variables. Model AIC (Akaike information criterion) is 227.3725.

[a]OR= Odds ratio, [b]CI= confidence intervals, [c]Ct load is defined as log-(e) omcB copies/swab.
