# Supplementary material for: Inverse relationship between microRNA-155 and -184 expression with increasing conjunctival inflammation during ocular Chlamydia trachomatis infection
Source: BMC Infect Dis. 2016 Feb 3;16:60. doi: 10.1186/s12879-016-1367-8 (PMC4739388; doi:10.1186/s12879-016-1367-8)
Supplement: Supplementary file 8 — Focused list of published roles for miR that are differentially expressed in follicular trachoma that relate to inflammation or fibrosis. (PDF 59 kb) [file 12879_2016_1367_MOESM8_ESM.pdf]

Additional file 8. Focused list of published roles for miR that are differentially expressed in TF that relate to inflammation or fibrosis.

miR	Expression in TF	Functions
**miR-155-5p**	Up-regulated	Hematopoiesis (1), regulation of T and B cell differentiation (2)<br>Essential for the development of Th1 and Th17 pathogen-specific immune responses (3)<br>Mainly pro-inflammatory, though some anti-inflammatory roles described<br>Induced upon exposure to TLR ligands or TNFα, dependent on transcription factors AP-1 and NFκB (4, 5)<br>Suppresses negative regulators of inflammation [56,57]
**miR-150-5p**	Up-regulated	Hematopoiesis - regulates T and B cell development (8)<br>Suppresses negative regulators of inflammation (SOCS1), promotes renal fibrosis (9, 10)
**miR-142-5p**	Up-regulated	Trans-activates miR-150, activates canonical Wnt pathway (11)
**miR-181b-5p**	Up-regulated	Endotoxin responsive, negatively regulates NFκB signaling (12)<br>Upregulated by TGFB1 in deep dermal fibroblasts → myofibroblast differentiation and hypertrophic scar formation [47]
**miR-181a-5p**	Up-regulated	Regulates lymphoid cell development, critical for T cell receptor selection in the thymus and follicular T helper cell differentiation [44,45]<br>Mediates TGFβ-induced epithelial-mesenchymal transition via repression of Smad7 in ovarian cancer [46]
**miR-342-3p**	Up-regulated	Overexpression inhibits cell proliferation, migration and invasion in cervical cancer (13)
**miR-184**	Down-regulated	Negatively regulates Wnt pathway (14), inhibits cell migration and proliferation (15)

**miR-4728-3p**	Down-regulated	Encoded in HER2 locus, targets estrogen receptor 1 alpha through a non-canonical target site (16)
**miR-132-3p**	Up-regulated	Endotoxin responsive, negatively regulates TLR-induced pro-inflammatory cytokine signaling (5, 17)
**miR-375**	Down-regulated	Supports differentiation of goblet cells (18)
**miR-10a-5p**	Up-regulated	Inhibits NFκB pro-inflammatory signaling pathway (19)
**miR-146b-3p**	Up-regulated	Endotoxin responsive, negatively regulates TLR-induced pro-inflammatory cytokine signaling (5)

References:

1. **Georgantas RW et al.** 2007. Proc Natl Acad Sci U S A **104**:2750–5.
2. **Turner M, Vigorito E**. 2008. Biochem Soc Trans **36**:531–3.
3. **Oertli M et al.** 2011. J Immunol **187**:3578–86.
4. **O'Connell RM et al.** 2007. Proc Natl Acad Sci U S A **104**:1604–9.
5. **Taganov KD, Boldin MP, Chang K-J, Baltimore D**. 2006. Proc Natl Acad Sci U S A **103**:12481–6.
6. **Pathak S et al.** Exp Mol Med **47**:e164.
7. **Wang P et al.** 2010. J Immunol **185**:6226–33.
8. **Zhou B et al.** 2007. Proc Natl Acad Sci U S A **104**:7080–5.
9. **Chen R-F et al.** 2014. J Infect **69**:366–74.

10. **Zhou H et al.** 2013. J Am Soc Nephrol **24**:1073–87.

11. **Isobe T et al.** 2014. Elife **3**:e01977.

12. **Sun X et al.** 2012. J Clin Invest **122**:1973–90.

13. **Li X-R et al.** 2014. FEBS Lett **588**:3298–307.

14. **Takahashi Y, Chen Q, Rajala RVS, Ma J-X**. 2015. FEBS Lett **589**:1143–9.

15. **Su Z et al.** 2015. Exp Ther Med **9**:961–966.

16. **Newie I et al.** 2014. PLoS One **9**:e97200.

17. **Shaked I et al.** 2009. Immunity **31**:965–73.

18. **Biton M et al.** 2011. Nat Immunol **12**:239–46.

19. **Fang Y et al.** 2010. Proc Natl Acad Sci U S A **107**:13450–5.
